# Porous Curdlan-Based Hydrogels Modified with Copper Ions as Potential Dressings for Prevention and Management of Bacterial Wound Infection—An In Vitro Assessment

**DOI:** 10.3390/polym12091893

**Published:** 2020-08-23

**Authors:** Aleksandra Nurzynska, Katarzyna Klimek, Iga Swierzycka, Krzysztof Palka, Grazyna Ginalska

**Affiliations:** 1 Chair and Department of Biochemistry and Biotechnology, Medical University of Lublin, Chodzki 1 Street, 20-093 Lublin, Poland; 59503@student.umlub.pl (A.N.); iga.swierzycka@interia.pl (I.S.); g.ginalska@umlub.pl (G.G.); 2 Faculty of Mechanical Engineering, Lublin University of Technology, Nadbystrzycka 26 Street, 20-618 Lublin, Poland; k.palka@pollub.pl

**Keywords:** antibacterial dressings, bacterial infections, chronic wounds, copper ions, cytotoxicity, glucans, fibroblasts, skin, wound healing

## Abstract

Bacterial infections at the wound site still remain a huge problem for current medicine, as they may lead to development of chronic wounds. In order to prevent such infections, there is a need to use wound dressings that possess ability to inhibit bacterial colonization. In this study, three new curdlan-based biomaterials modified with copper ions were fabricated via simple and inexpensive procedure, and their structural, physicochemical, and biological properties in vitro were evaluated. Received biomaterials possessed porous structure, had ability to absorb high amount of simulated wound fluid, and importantly, they exhibited satisfactory antibacterial properties. Nevertheless, taking into account all evaluated properties of new curdlan-based biomaterials, it seems that Cur_Cu_8% is the most promising biomaterial for management of wounds accompanied with bacterial infections. This biomaterial exhibited the best ability to reduce *Escherichia coli* and *Staphylococcus aureus* growth and moreover, it absorbed the highest amount of simulated wound fluid as well as enabled optimal water vapor transmission. Furthermore, Cur_Cu_8% biomaterial possessed the best values of selective indexes, which determine its potential safety in vitro. Thus, Cur_Cu_8% hydrogel may be considered as a promising candidate for management of infected wounds as well as it may constitute a good platform for further modifications.

## 1. Introduction

Microbial growth is considered as a crucial factor, which affects the rate of wound healing [1,2,3,4]. Development of bacterial infections at the wound site (especially caused by *Staphylococcus aureus*, *Enterococcus faecalis*, *Pseudomonas aeruginosa*, and *Escherichia coli*) leads to prolonged inflammation stage, which does not allow for proper wound healing and also may lead to sepsis or even to patient dead [1,4,5,6,7].

Prevention of bacterial colonization as well as management of bacterial infections at the wound site require the use of appropriate dressings. Such biomaterials must primarily be non-toxic, non-allergenic, and non-adherent. Moreover, they should be semi-permeable for water and oxygen, should absorb wound exudate, and maintain moist environment at the wound site. Most important, they must prevent bacterial colonization [1,3,6,8,9,10]. Thus, advanced dressings are frequently improved with antimicrobial agents such as antibiotics [8,11,12], antiseptics [11,13,14], and metals [11,15,16,17]. The modification of dressings with metal ions (e.g., silver, zinc, and copper) constitutes promising approach towards management of infected wounds, especially resulted from colonization of antibiotic resistant bacterial strains [15,16,17,18]. Among mentioned metal ions, copper has had great attention due to its crucial role in wound healing. It exhibits not only a broad spectrum of antimicrobial activity but also enhance wound healing via promotion of angiogenesis as well as supporting of expression and stabilization of extracellular skin proteins [7,18]. It is also worth noting that Cu^2+^ is cheaper, more oxidation resistant, and more stable in comparison with Ag^+^ [19]. Thus to date, many wound dressings enriched with copper ions have been developed and described in literature [10,15,16,19,20,21]. For instance, Basu et al. [15] fabricated wood-derived nanocellulose hydrogels modified with copper ions and demonstrated that such biomaterials may be considered as promising wound dressings, as they possessed antibacterial activity against both *Staphylococcus epidermidis* and *Pseudomonas aeruginosa*. Klinkajon and Supaphol [16] showed that alginate hydrogels cross-linked by copper ions possessed good ability to absorb fluids, had hemostatic properties as well as enabled water vapor transmission. Moreover, some of biomaterials exhibited antibacterial activity against *Staphylococcus aureus*, *Staphylococcus epidermidis*, *Streptococcus pyogenes*, and *Escherichia coli.* Li et al. [19] presented chitosan-Cu^2+^/NH_3_ physical wound dressings and they proved that such biomaterials possessed weak antibacterial activity against *Enterococcus faecalis*, *Escherichia coli* as well as *Staphylococcus aureus*, but caused strong inhibition of *Pseudomonas aeruginosa* growth. Meanwhile, Ren et al. [21] fabricated chitosan film enriched with copper metal-organic framework and proved its antibacterial activity in vitro (study on *Staphylococcus aureus* and *Escherichia coli*) as well as in vivo (study on Balb/c mice infected by *Staphylococcus aureus*).

The aim of this study was to determine potential application of curdlan-based hydrogels enriched with copper ions as dressings for prevention of bacterial colonization and for management of infected wounds. Curdlan (linear β-1,3-glucan) is a non-toxic polymer, synthesized by *Agrobacterium* sp. This polysaccharide and its derivatives possess wide spectrum of biological actions including immunomodulatory, anti-cancer, anti-coagulant, and anti-infective properties [22]. Furthermore, it has ability to form hydrogel during heat-treatment process (incubation temperature above 55 °C) as well as in the presence of divalent ions (such as calcium, copper or zinc) [23,24]. Thus, thanks to its beneficial properties, curdlan is willingly used for many medical and pharmaceutical applications [22,25,26]. Nevertheless, to our best knowledge, the biomedical potential of curdlan biomaterial enriched with copper ions has not yet been investigated.

In this study, novel curdlan-based biomaterials were fabricated by cost-effectiveness, simple as well as relatively rapid procedure, which involved ion-exchanging dialysis against copper ions followed by freezing at −80 °C, and freeze-drying. In assumption, combination of these fabrication techniques should allow to obtain biomaterials, which will have porous structure, will absorb great amount of liquids, and most important will possess antibacterial properties, thanks to copper content. To establish so, structural, physicochemical, antibacterial as well as cytotoxic properties of curdlan-based hydrogels were evaluated.

## 2. Materials and Methods

### 2.1. Materials

Bovine serum albumin (BSA), Live/Dead Cell Double Staining Kit, penicillin-streptomycin solution, phosphate buffered saline (PBS), silica gel with indicator (orange gel), sodium dodecyl sulfate (SDS), thiazolyl blue tetrazolium bromide (MTT), tris-hydroxymethyl aminomethane (TRIS), and trypsin-EDTA solution (0.25%) were obtained from Sigma-Aldrich Chemicals, Poznań, Poland. Curdlan (MW 80 kDa) was purchased from Wako pure Chemicals Industries, Osaka, Japan, whereas fetal bovine serum (FBS) from Pan-Biotech, Aidenbach, Germany. Eagle’s Minimum Essential Medium (EMEM), normal human skin fibroblasts (BJ cell line, CRL-2522^TM^), *Staphylococcus aureus* (ATCC 25923) as well as *Escherichia coli* (ATCC 25922) were supplied by ATCC, London, UK, while Mueller-Hinton agar (MHA) and Mueller-Hinton broth (MHB) by Oxoid, UK. Live/dead BacLight Bacterial Viability Kit was supplied by Invitrogen, Poland, while Viabank^TM^ vials by Medical Wire & Equipment, Corsham, UK. Calcium chloride (CaCl_2_), copper (II) chloride (CuCl_2_), sodium chloride (NaCl), hydrochloric acid (HCl), and sodium hydroxide (NaOH) were purchased from Avantor Performance Materials, Gliwice, Poland. In turn, copper ions detection kit was obtained from BioMaxima, Lublin, Poland.

### 2.2. Fabrication of Curdlan-Based Hydrogels Modified with Copper Ions

The curdlan-based biomaterials were prepared during inexpensive and simple procedure that includes combination of two techniques, i.e., ion-exchanging dialysis against copper ions and freeze-drying. Firstly, the solutions composing of 11 wt.% of curdlan in 0.3 M aqueous NaOH were obtained, and then they were transferred into forms (round-shaped with 2.2 cm in diameter). Subsequently, these solutions were subjected to ion-exchanging dialysis against copper chloride solutions at concentration of 4%, 6%, and 8%. After 24-h incubation at 25 °C, solid biomaterials were rinsed three times with deionized water, blotted with tissue paper, and put into freezer at −80 °C for 2 days. In order to obtain dry samples, frozen samples were freeze-dried for 24 h (LYO GT2-Basic, SRK Systemtechnik GmbH, Riedstadt, Germany). The obtained samples were marked as Cur_Cu_4%, Cur_Cu_6%, and Cur_Cu_8% (Table 1). All samples were sterilized by ethylene oxide.

### 2.3. Assessment of Morphology of the Biomaterials

The morphology of the biomaterials was evaluated using scanning electron microscopy (Nova NanoSEM 450, FEI, Oxford, UK), equipped with Octane Pro EDS detector (EDAX), which allowed for chemical analysis and identification of elements present on biomaterial surface.

### 2.4. Evaluation of Ability of Biomaterials to Absorb Wound Exudate

This experiment was performed according to guidelines described by Rezvanian et al. [27]. Prior to experiment, the simulated wound fluid (SWF) was prepared. To obtain 1 L of SWF, the following components were dissolved in deionized water: 2.22 g of CaCl_2_, 23.38 g of NaCl, 9.69 g of TRIS, and 20 g of BSA. The pH of prepared solution was adjusted to 7.5. Then, three independent biomaterial samples (*n = 3*) were weighted, and soaked in simulated wound fluid (SWF) at room temperature. After appropriate time period, biomaterials were removed from SWF, blotted with tissue paper, weighted, and re-immersed in solution. The experiment was carried out for 24 h. The ability of biomaterials to swell in SWF was expressed as swelling ratio that was calculated as follows:

(1) $$\mathrm{SW}\;\left(\%\right)=\frac{\left(\mathrm{Ws}-\mathrm{Wd}\right)}{\mathrm{Wd}}\times \;100$$

where Ws is a weight of wet biomaterials and Wd is a weight of dry ones.

### 2.5. Evaluation of Water Vapor Transmission Rate

Ability of biomaterials to transmit water vapor was evaluated according to procedure described by Rezvanian et al. [28] with some modifications. Prior to test, 5 g of silica gel with indicator was put into glass vials (diameter of mouth of vial was 1 cm) and dried for 24 h at 60 °C (Drying oven SUP-65, Wamed). At the same time, three independent biomaterial samples (*n* = 3) were immersed in SWF (24 h, room temperature) in order to obtain totally soaked samples. Then, the vials (with dry silica gel) were weighted. The biomaterials were taken out of solution, blotted with tissue paper, and precisely mounted on the mouth of vials using rubber rings to prevent any water vapor influx through the boundary. The vials with biomaterials were transferred to the incubator (Heraeus cytoperm 2, Thermo Scientific, Waltham, MA, USA) for 24 h (37 °C, 95% relative humidity). After this time, the biomaterials from vials were removed and the vials with wet silica gel were weighted. The water vapor transmission rate (WVTR) was calculated using the following formula:

(2) $$\mathrm{WVTR}\;\left(g/m^{2}/\mathrm{day}\right)=\frac{\mathrm{Ww}-\mathrm{Wd}}{S}$$

where Ww is a weight of vial with wet silica gel (after 24-h incubation), Wd is a weight of vial with dry silica gel (before incubation), and S is surface area of mouth of vial (m^2^).

### 2.6. Measurement of Concentration of Copper Ions Released from Biomaterials

The ability of biomaterials to release copper ions into an aqueous environment was assessed using two culture media, i.e., Mueller-Hinton broth (MHB) and Eagle’s Minimum Essential Medium (EMEM), which are dedicated for culture of bacteria or skin fibroblasts, respectively. For this purpose, three independent biomaterial specimens (*n* = 3) were weighted, placed into 12-well plate, and incubated with MHB or EMEM at 37 °C for 24 h. The extraction ratio was 0.1 g of biomaterial per 1 mL of appropriate medium. This proportion is recommended by ISO 10993-5: 2009 standard [29]. In order to obtain control extracts, MHB or EMEM were incubated without biomaterials. The concentration of copper ions in collected liquid extracts was assessed using copper ions detection kit, according to manufacturer protocol. This kit based on direct colorimetric method using 3,5-DiBr-PAESA (4-(3,5-dibromo-2-pyridylazo)-N-ethyl-N-sulfopropylaniline monosodium salt, monohydrate). The copper ions react with 3,5-DiBr-PAESA and form stable blue complex. The absorbance of this complex was measured at 582 nm and it is proportional to the concentration of copper in the sample.

### 2.7. Antibacterial Activity of Biomaterials

The bacterial strains were stored at −70 °C in Viabank^TM^ vials. Before each experiments, the bacteria—*Staphylococcus aureus* (*S. aureus*) and *Escherichia coli* (*E. coli*) were subcultured on MHA plates at 37 °C for 24 h. Afterwards, bacterial colonies were suspended in MHB. The bacterial suspensions were adjusted to the turbidity of a 0.5 McFarland standard (PhoenixSpec Nephelometer, Becton Dickinson, Franklin Lakes, NJ, USA).

#### 2.7.1. Determination of Zones of Bacterial Growth Inhibition

In order to assess the preliminary antibacterial activity of investigated biomaterials, the disc diffusion method was applied [30]. For this purpose, prepared bacterial inoculums (*S. aureus* or *E. coli*) were spread over the Petri Dishes containing MHA using sterile cotton swabs. Then, biomaterial specimens were placed onto MHA with inoculated bacteria and such Petri Dishes were transferred to the incubator (37 °C, 24 h). After this time, the zones of bacterial growth inhibition around the biomaterial samples were measured. The experiment was carried out in three replicates (*n* = 3).

#### 2.7.2. Evaluation of Inhibition of Bacterial Growth in Direct Contact with Biomaterials

Prior to experiment, six independent biomaterial samples (*n* = 6) were placed in 12-well plates. Then, 2 mL of the appropriate bacterial suspension (*S. aureus* or *E. coli*) was added directly to each biomaterial samples. The bacterial inoculates added to wells without biomaterials were served as controls. The plates were incubated at 37 °C for 24 h. The inhibition of bacterial growth in the direct contact with biomaterials was assessed qualitatively. Therefore, after incubation time, the bacterial suspensions from the wells without biomaterials (control) or with biomaterials were collected, centrifuged (13,000 rpm, 10 min), suspended in 40 μL of fresh MHA, and stained with Live/dead BacLight Bacterial Viability Kit according to manufacturer procedure. Briefly, this kit is composed of two fluorescent dyes, i.e., SYTO 9 and propidium iodide. Firstly, stock solution of dyes in 0.9% NaCl was prepared (1 μL of SYTO 9 + 1 μL of propidium iodide + 8 μL of NaCl solution). Then, 1 μL of such stock solution was added to 40 μL of bacterial suspension. After 15 min. incubation at 37 °C, the dyed bacteria were transferred on glass microscope slides and observed under confocal laser scanning microscope (CLSM, Olympus Fluoview equipped with FV1000, Olympus, Tokyo, Japan Manufacturer, City, Country). The excitation/emission maxima for SYTO 9 and propidium iodide are about 480/500 nm and 490/638 nm, respectively. Thus, live bacteria (stained with SYTO 9) emitted green fluorescence, whereas dead ones (stained with propidium iodide) emitted red fluorescence.

#### 2.7.3. Evaluation of Inhibition of Bacterial Growth in Indirect Contact with Biomaterials–Test on Extracts

This experiment was carried out according to general procedure described by Siek et al. [31], but with some modifications. Thus, the influence of fluid extracts obtained from the biomaterials (*n* = 3) on bacterial growth was investigated. Such extracts were prepared using MHB as described in Section 2.6. Received extracts (100%) were then diluted in fresh MHB (ranged from 90–10%) in order to mimic experiment conditions, which were applied during cytotoxicity evaluation (Section 2.8). Then, 100 μL of appropriate extracts (100–10%) were placed into 96-well plates and 10 μL of bacterial inoculates (*S. aureus* or *E. coli*) were added. As a control extract, pure MHA incubated without biomaterials was used. After 24-h incubation, 100 μL of bacterial suspensions (three repetitions of each groups) were seeded on Petri Dishes containing MHA, and the plates were transferred into incubator (37 °C). On the next day, bacterial colonies were counted and the results were expressed as a % of log_10_ CFU/mL of control bacteria. Based on these data, values of concentration of extract (CE_50_) were calculated via 4-parameter nonlinear regression analyses (GraphPad Prism 5, Version 5.04, GraphPad, San Diego, CA, USA). The CE_50_ means concentration of extract required for reduction of *S. aureus* or *E. coli* growth to 50%.

### 2.8. Cytotoxicity Evaluation and Determination of Selective Index

Cytotoxic activity of tested biomaterials was evaluated by indirect method, i.e., using fluid extracts obtained from biomaterials (*n* = 3). Firstly, the BJ cells were seeded in 96-well plates in 100 μL of EMEM at concentration of 1.5 × 10^5^ cells/mL and incubated at 37 °C for 24 h. At the same time, liquid extracts from biomaterials were prepared in EMEM as described in Section 2.6. Obtained extracts (100%) were further diluted in fresh EMEM (ranged from 90–10%). As a control extract, pure EMEM incubated without biomaterials was used. Then, culture medium was gently removed and 100 μL of appropriate extracts (100–10%) or control extract were added to the BJ cells. After 24-h incubation, cell viability was assessed both quantitatively (via MTT assay) and qualitatively (via cell staining with Live/Dead Cell Double Staining Kit). The MTT test was performed according to procedure described earlier [32]. Received results were expressed as % of viability of control cells. Based on these data, values of CE_50_ were calculated (4-parameter nonlinear regression analyses, GraphPad Prism 5, version 5.04). The CE_50_ means concentration of extract required for reduction of viability of BJ cells to 50%. In turn, values of selective index (SI) were calculated as follows:

(3) $$\mathrm{SI}=\;\frac{{\mathrm{CE}}_{50}\;\mathrm{for}\;\mathrm{fibroblast}\;\mathrm{cells}}{{\mathrm{CE}}_{50}\;\mathrm{for}\;\mathrm{bacterial}\;\mathrm{cells}}$$

The BJ cell viability after staining with Live/Dead Cell Double Staining Kit (in accordance with manufacturer protocol) was assessed using CLSM (Olympus Fluoview equipped with FV1000). Live fibroblasts emitted green fluorescence, whereas dead ones emitted red fluorescence.

### 2.9. Statistical Analysis

All experiments were performed using at least three independent biomaterial samples and the obtained results were presented as mean values ± standard deviation (SD). In order to determine the statistically significant differences between groups (*p* < 0.05), one-way ANOVA test followed by Tukey’s multiple comparison test was performed using GraphPad Prism 5, Version 5.04 Software.

## 3. Results and Discussion

### 3.1. Morphology of Biomaterials

**Figure 1:**
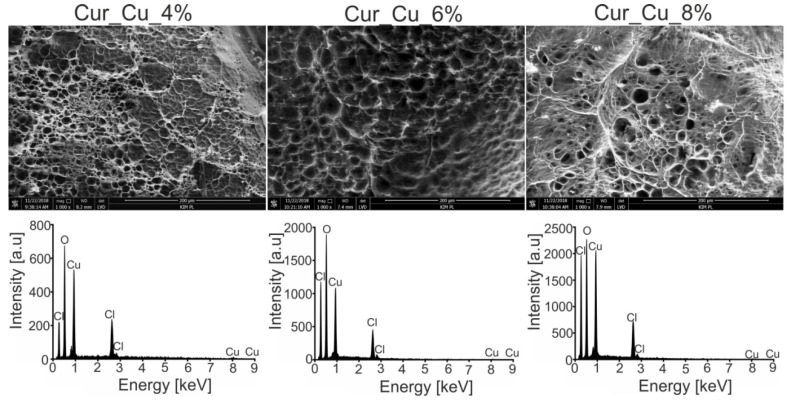
Scanning electron microscopy (SEM) images and energy dispersive spectroscopy (EDS) spectra of Cur_Cu_4%, Cur_Cu_6%, and Cur_Cu_8%. Magnification of SEM images: 1000×.

Performed SEM analysis revealed that all biomaterials possessed rough and porous structure (Figure 1). Porous structure is considered as an important property of biomaterial because it significantly increases its specific surface area. It was demonstrated that the higher specific surface area is, the biomaterial has better absorbent properties [33]. Therefore, it seems that novel porous curdlan-based biomaterials should exhibit good ability to swell in the contact with liquids. In turn, EDS spectra (Figure 1) revealed that biomaterial surfaces were rich in copper precipitates, what confirms that dialysis method against CuCl_2_ solution enables successful incorporation of copper into curdlan-based biomaterials. Taking together, it is worth to underline that combination of two techniques, i.e., ion exchanging dialysis followed by freezing, and freeze-drying allows to obtain porous biomaterials which are enriched with copper.

### 3.2. Absorption Capacity of Biomaterials

**Figure 2:**
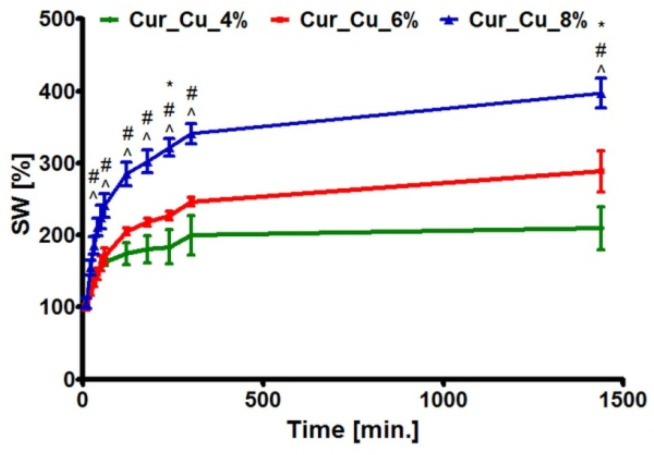
The ability of Cur_Cu_4%, Cur_Cu_6%, and Cur_Cu_8% samples to swell in simulated wound fluid during 24-h incubation. #—significantly different results between Cur_Cu_6% and Cur_Cu_8%, ^—significantly different results between Cur_Cu_4% and Cur_Cu_8%, *—significantly different results between Cur_Cu_4% and Cur_Cu_6%; one-way ANOVA followed by Tukey’s multiple comparison test, *p* < 0.05.

The test carried out in SWF demonstrated that throughout the experiment period, Cur_Cu_8% absorbed the highest amount of liquid, whereas the amount of SWF absorbed by Cur_Cu_4% was the slightest (Figure 2). At the end of test (after 24-h incubation), the swelling ratios for biomaterials were 209.60 ± 29.99% (Cur_Cu_4%), 288.80 ± 29.99% (Cur_Cu_6%), and 397.20 ± 20.80% (Cur_Cu_8%). The suitable dressing for chronic wounds should absorb high amount of exudate, which can achieve even 12 L/m^2^ [34]. When compared investigated curdlan-based dressings with others biomaterials dedicated for treatment of chronic wounds, it is clear that newly fabricated biomaterials have good ability to absorb liquids. For instance, the agar-sericin hydrogel film dressings dedicated for chronic wounds possessed swelling ratios that were around 320% [35], while the ability of alginate-pectin hydrogel films to absorb SWF was slightly above 250% [27]. Thus, these results indicate that porous curdlan-based biomaterials, especially Cur_Cu_6% as well as Cur_Cu_8% exhibit optimal liquid absorption capacity for wounds with moderate to high exudate levels.

### 3.3. Water Vapor Transmission Rate of Biomaterials

**Figure 3:**
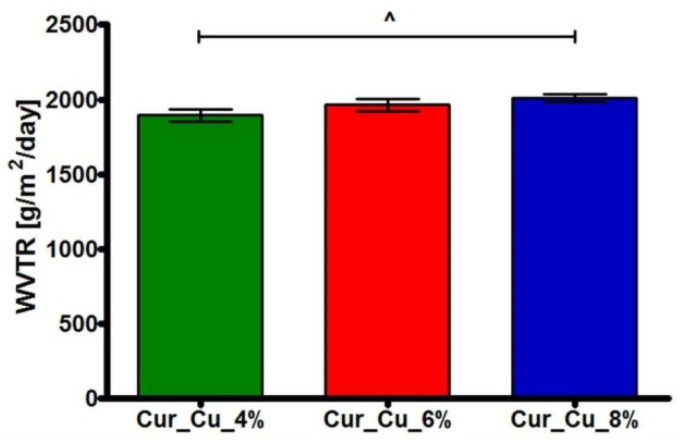
The ability of Cur_Cu_4%, Cur_Cu_6%, and Cur_Cu_8% samples to transmit water vapor during 24-h experiment. ^—significantly different results between Cur_Cu_4% and Cur_Cu_8%; one-way ANOVA followed by Tukey’s multiple comparison test, *p* < 0.05.

Water vapor transmission test (Figure 3) showed that fabricated curdlan-based biomaterials possessed WVTR close to approx. 1894 g/m^2^/day (Cur_Cu_4%), 1964 g/m^2^/day (Cur_Cu_6%), and 2009 g/m^2^/day (Cur_Cu_8%). According to available data, the appropriate dressings should inhibit excessive dehydration of skin, but they should also enable skin to “breath”. For these reasons, the WVTR for dressings should achieve 2000–2500 g/m^2^/day [27,36]. Thus, among obtained samples, only Cur_Cu_8% biomaterial exhibited optimal value of WVTR (above 2000 g/m^2^/day).

### 3.4. Ability of Biomaterials to Release Copper Ions

**Figure 4:**
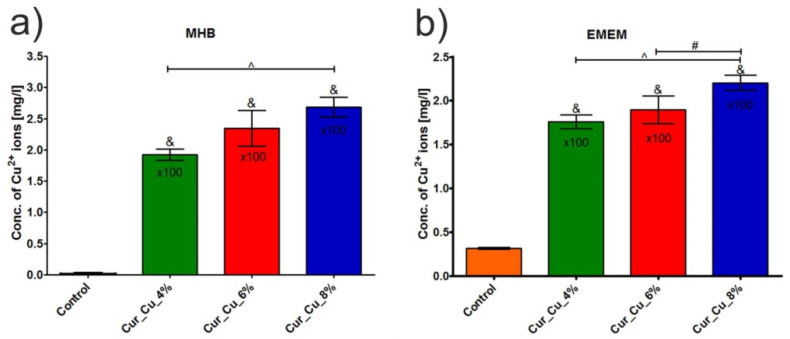
Concentration of copper ions in extracts obtained from Cur_Cu_4%, Cur_Cu_6%, and Cur_Cu_8% biomaterials after 24-h incubation in culture media: (**a**) Mueller-Hinton broth (MHB) and (**b**) Eagle’s Minimum Essential Medium (EMEM). &—significantly different results compared to control (media incubated without biomaterials), #—significantly different results between Cur_Cu_6% and Cur_Cu_8%, ^—significantly different results between Cur_Cu_4% and Cur_Cu_8%; one-way ANOVA followed by Tukey’s multiple comparison test, *p* < 0.05.

The release test showed that concentration of copper ions in extracts obtained from all tested biomaterials was significantly higher (*p* < 0.05) compared to concentration of these ions in control extracts–media incubated without biomaterials (Figure 4a,b). Among tested dressings, Cur_Cu_8% possessed the greatest ability to release copper ions. Thus, after 24-h incubation in MHB or EMEM, the concentration of copper ions released from Cur_Cu_8% was 268.30 ± 15.84 mg/L (Figure 4a) and 220.50 ± 10.82 mg/L (Figure 4b), respectively. It is worth to note that applied culture medium (MHB or EMEM) did not significantly affect ability of biomaterials to release copper ions, what is a crucial for reliable evaluation of antibacterial and cytotoxic activities of biomaterials.

### 3.5. Antibacterial Activity of Biomaterials

**Figure 5:**
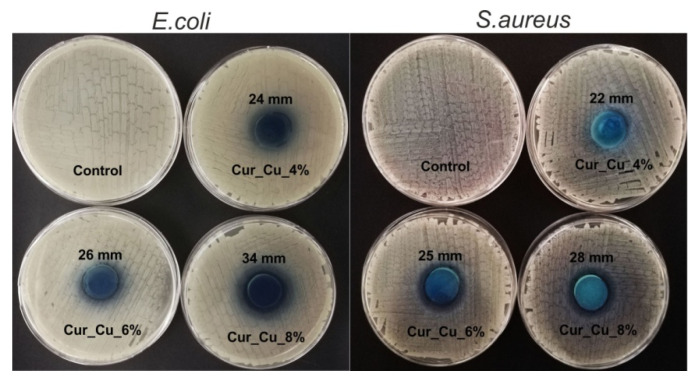
Representative images presenting zones of *E. coli* and *S. aureus* growth inhibition around Cur_Cu_4%, Cur_Cu_6%, and Cur_Cu_8% biomaterials. The diameter of samples was equal to 22 mm.

Preliminary antibacterial test (disc diffusion method) demonstrated that all biomaterials possessed ability to inhibit *E. coli* and *S. aureus* growth (Figure 5). Nevertheless, it was observed that antibacterial properties of biomaterials increased in the following trend: Cur_Cu_4% < Cur_Cu_6% < Cur_Cu_8%. Thus, the greatest zones of bacterial growth inhibition (34 mm for *E. coli* and 28 mm for *S. aureus*) were observed for Cur_Cu_8%, what most likely resulted from its the best ability to release copper ions (Figure 4).

**Figure 6:**
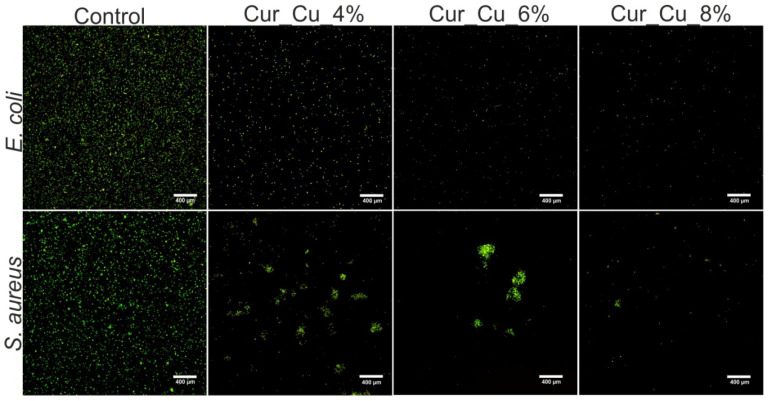
Representative images showing live (green fluorescence) bacteria—*E. coli* and *S. aureus*—after 24-h incubation with Cur_Cu_4%, Cur_Cu_6%, and Cur_Cu_8% biomaterials. The bacteria incubated without biomaterials were served as control of experiment. In all tested groups, none dead cells (red fluorescence) were visible. Magnification 400×, scale bar equals 400 μm.

In the next step, the ability of biomaterials to inhibit bacterial growth in liquid medium (MHB) was evaluated via cell staining with Live/dead BacLight Bacterial Viability Kit. Performed microscope observations (Figure 6) clearly confirmed the results obtained during screening antibacterial test (Figure 5), indicating that all dressings had antibacterial activity. After 24-h incubation with biomaterials, the number of *E. coli* as well as *S. aureus* was significantly lower compared to number of control bacterial cells (cell incubated without biomaterials) (Figure 6). Despite the visible reduction of amount of viable bacteria after direct contact with biomaterials, none dead bacterial cells were observed, what indicates that Cur_Cu_4%, Cur_Cu_6%, and Cur_Cu_8% possess bacteriostatic properties but not bactericidal ones.

**Figure 7:**
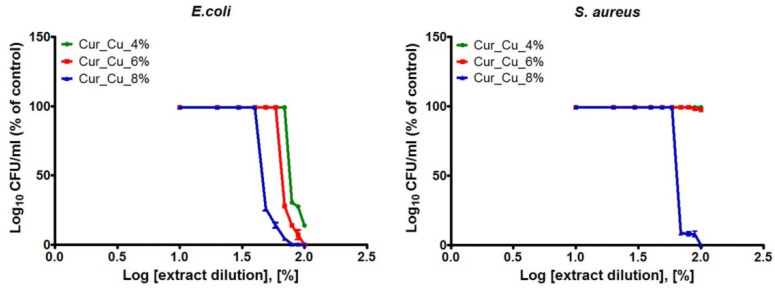
The antibacterial activity of extracts obtained from Cur_Cu_4%, Cur_Cu_6%, and Cur_Cu_8% biomaterials. The initial extracts (100%) were diluted in fresh MHB (90–10%) in order to determine bacterial response to extract solutions at different concentrations.

The last experiment included evaluation of antibacterial properties of extracts obtained from biomaterials. All extracts obtained from tested biomaterials exhibited greater ability to reduce number of *E. coli* compared to *S. aureus* (Figure 7)*,* what clearly proved the results obtained during previous experiments (Figure 5). It was also demonstrated that extracts obtained from Cur_Cu_8% possessed the best ability to inhibit both *E. coli* and *S. aureus* growth (Figure 7).

Based on antibacterial results obtained for different concentrations of extracts (Figure 7), the values of CE_50_ were calculated (Table 2). It was demonstrated that extracts obtained from Cur_Cu_4% and Cur_Cu_6% possessed moderate activity against *E. coli* (CE_50_ close to 76% and 67%, respectively), while they did not reduce *S. aureus* growth. In turn, extracts obtained from Cur_Cu_8% possessed the best ability to inhibit bacterial growth, with CE_50_ values equal to 46.65 ± 2.21% (*E. coli*) and 65.86 ± 2.12% (*S. aureus*). These results indicated that not only direct contact with Cur_Cu_8% biomaterial provide antibacterial protection, but also released from biomaterial copper ions possess ability to reduce number of bacterial cells.

Taking together, three independent experiments: disc diffusion test, test in direct contact, and test on extracts clearly showed that Cur_Cu_8% biomaterial possessed the most beneficial, antibacterial properties. In many research studies, antibacterial properties of biomaterials have been assessed only via disc diffusion method. It is well known, that chronic wounds are associated with high amount of exudate. Thus, released antibacterial agents from biomaterial may be diluted by secreted exudate. For this reason, it is crucial to determine whether dilution of antibacterial agents allows to preserve antibacterial protection. Our research proved that Cur_Cu_8% dressing as well as released by it copper ions should provide antibacterial protection during management of wounds with moderate to high exudate levels.

### 3.6. Cytotoxic Activity of Biomaterials

**Figure 8:**
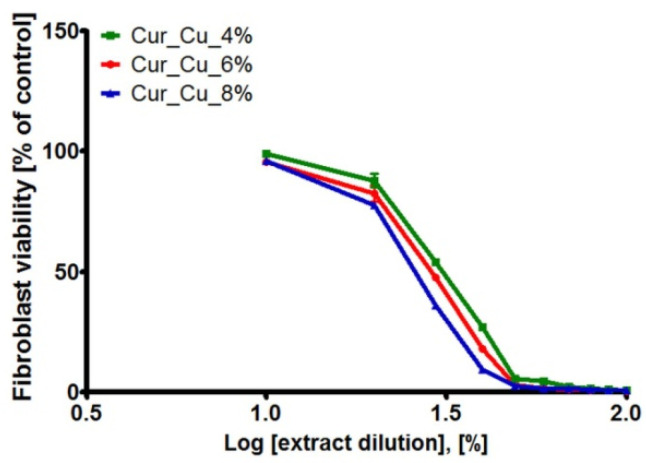
The cytotoxic activity of extracts obtained from Cur_Cu_4%, Cur_Cu_6%, and Cur_Cu_8% biomaterials. The initial extracts (100%) were diluted in fresh Eagle’s Minimum Essential Medium (EMEM) (90–10%) in order to determine skin fibroblast (BJ cell line) response to extract solutions at different concentrations.

**Figure 9:**
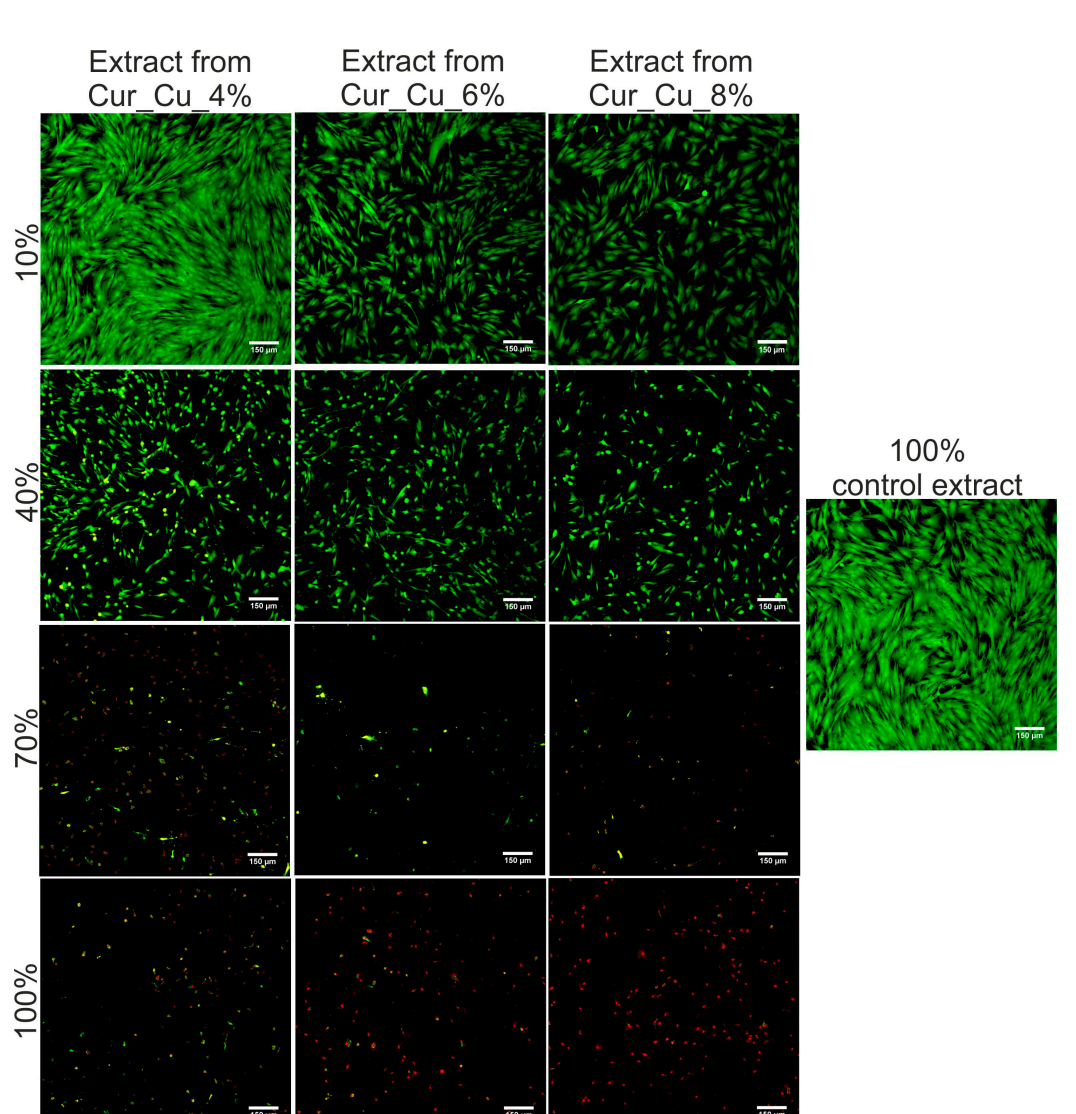
Representative images showing live (green fluorescence) and dead (red fluorescence) BJ cells after 24-h incubation with different concentration of extracts from Cur_Cu_4%, Cur_Cu_6%, and Cur_Cu_8% biomaterials. Pure culture medium incubated without biomaterials was served as control extract. Magnification 100×, scale bar equals 150 μm.

The MTT assay (Figure 8) as well as Live/Dead Cell Double Staining Kit (Figure 9) revealed that initial extracts obtained from all tested biomaterials were highly toxic towards normal human skin fibroblasts. After 24-h incubation with extracts (100%), BJ cell viability was approximately 0.5%. It was also observed that cells were mostly dead (emitting red fluorescence) and only single live cells (emitting green fluorescence) were visible (Figure 9). Dilution of extracts caused that viability of BJ cells significantly increased (Figure 8 and Figure 9).

According to ISO recommendations [29], if initial extract (100%) reduces cell viability below 70% compared to control, it should be diluted and its concentration required to inhibit cell viability to 50% (CE_50_) should be determined. Thus, the values of CE_50_ for BJ cells were calculated and summarized in Table 3. It was shown that extracts from tested biomaterials exhibited cytotoxic activity, with CE_50_ ranging 26–30%. Nevertheless, in order to determine which biomaterial seems to be the most safe in vitro, the SI values were calculated (Table 3). Obtained results clearly demonstrated that among tested samples, Cur_Cu_8% biomaterial exhibits the best SI values (0.56 and 0.40).

Due to high cytotoxicity in vitro of fabricated biomaterials, their biomedical potential as wound dressings seems to be limited. Biocompatibility of biomaterial is a mandatory feature, which allows to consider its potential medical applications [6,23]. It is worth to underline that biomaterials enriched by metal ions most often exhibit antimicrobial activity, but also they are very toxic towards eukaryotic cells [37]. Received Cur_Cu_4%, Cur_Cu_6%, and Cur_Cu_8% biomaterials possessed good antibacterial activity, so it was expected that they also can be cytotoxic. For these reasons, additional in vivo research are necessary to precise determination of toxic activity of these biomaterials. Moreover, obtained results suggest that there is a need to develop modified curdlan-based dressings, which will have antibacterial properties and will not significantly decrease fibroblast viability. One interesting way includes modification of biomaterials with metal ions combined with plant extracts [38]. Thus, in future our research will be focused on modification of curdlan-based biomaterials with metal ions important for wound healing (e.g., Cu^2+^, Zn^2+^) in combination with antimicrobial and non-toxic plant extracts.

## 4. Conclusions

In this study, curdlan-based biomaterials modified by copper ions were fabricated via an inexpensive as well as a relatively simple procedure, which involved ion-exchanging dialysis followed by freezing and freeze-drying. Received biomaterials possessed porous structure (as proven by SEM analysis), exhibit good ability to absorb simulated wound fluid, and had capacity to release a high amount of copper ions to the aqueous environment. Among tested samples, Cur_Cu_8% had the best physicochemical and antibacterial properties, as it enabled water vapor transmission on optimal level and significantly reduces *E. coli* and *S. aureus* growth. Moreover, comparison of antibacterial and cytotoxic activities (expressed as selective indexes), indicated that of Cur_Cu_8% biomaterial exhibited the highest in vitro safety compared to other samples.

## Figures and Tables

**Table 1:** Composition of novel curdlan-based hydrogels modified with copper ions.

Sample Code	Concentration of Curdlan in 0.3 M NaOH Solution (wt.%)	Concentration of Copper Chloride Solution Used for Ion-Exchanging Dialysis (%)
Cur_Cu_4%	11	4
Cur_Cu_6%	11	6
Cur_Cu_8%	11	8

**Table 2:** The concentration of extract (CE_50_) obtained from Cur_Cu_4%, Cur_Cu_6%, and Cur_Cu_8% biomaterials, which is required to inhibit *E. coli* or *S. aureus* growth to 50%. The CE_50_ values were calculated via 4-parameter nonlinear regression analyses, GraphPad Prism 5, version 5.04).

Biomaterial Used to Obtain Extract in Mueller-Hinton Broth (MHB)	Concentration of Extract Required to Inhibit Bacterial Growth to 50% (CE_50_) [%]	
	*E. coli*	*S. aureus*
Cur_Cu_4%	76.48 ± 1.45	ND *
Cur_Cu_6%	66.73 ± 2.34	ND *
Cur_Cu_8%	46.65 ± 2.21	65.86 ± 2.12

* ND—not determined. All tested concentrations of extract did not exhibit ability to inhibit bacterial growth to 50%.

**Table 3:** The concentration of extract (CE_50_) obtained from Cur_Cu_4%, Cur_Cu_6%, and Cur_Cu_8% biomaterials, which is required to inhibit fibroblast viability (BJ cells) to 50%. The CE_50_ values were calculated via 4-parameter nonlinear regression analyses, GraphPad Prism 5, version 5.04). The values of selective index were calculated based on results obtained for bacteria and fibroblasts. The higher value of Selective Index (SI) is, the extract from biomaterial is safer for eukaryotic cells.

Biomaterial Used to Obtain Extract in Eagle’s Minimum Essential Medium (EMEM)	Concentration of Extract Required to Inhibit Fibroblast Viability to 50% (CE_50_) [%]	Selective Index (SI) * $\mathrm{SI}=\;\frac{{\mathrm{CE}}_{50}\;\mathrm{for}\;\mathrm{fibroblast}\;\mathrm{cells}}{{\mathrm{CE}}_{50}\;\mathrm{for}\;\mathrm{bacterial}\;\mathrm{cells}}$	
		*E. coli*	*S. aureus*
Cur_Cu_4%	30.91 ± 1.23	0.40	ND **
Cur_Cu_6%	29.49 ± 1.43	0.44	ND **
Cur_Cu_8%	26.48 ± 1.73	0.56	0.40

* The values of CE_50_ for bacterial cells were adopted from Table 2. ** ND—not determined. It was not possible to obtain SI value due to lack of value of CE_50_ for bacterial cells.
